# The effect of static versus dynamic stimuli on visual processing of sexual cues in androphilic women and gynephilic men

**DOI:** 10.1098/rsos.172286

**Published:** 2018-06-13

**Authors:** Samantha J. Dawson, Meredith L. Chivers

**Affiliations:** Department of Psychology, Queen's University at Kingston, Ontario, Canada

**Keywords:** eye tracking, attention, sexual interest, sexual arousal, gender difference, gender-specificity

## Abstract

Models of sexual response posit that attentional processing of sexual cues is requisite for sexual responding. Despite hypothesized similarities in the underlying processes resulting in sexual response, gender differences in sexual arousal patterns are abundant. One such gender difference relates to the stimulus features (e.g. gender cues, sexual activity cues) that elicit a response in men and women. In this study, we examined how stimulus modality (static visual images versus dynamic audiovisual films) and stimulus features (gender, sexual activity and nonsexual contextual cues) influences attentional (i.e. gaze) and elaborative (i.e. self-reported attraction (SRA), self-reported arousal) processing of sexual stimuli. Men's initial and controlled attention was consistently gender-specific (i.e. greater attention towards female targets), and this was not influenced by stimulus modality or the presence of sexual activity cues. By contrast, gender-specificity of women's attention patterns differed as a function of attentional stage, stimulus modality and the features within the stimulus. Degree of specificity was positively predictive of SRA in both genders; however, it was not significantly predictive of self-reported arousal. These findings are discussed in the context of gendered processing of visual sexual information, including a discussion of implications for research designs.

## 1. Introduction

According to early and contemporary models, sexual arousal is the result of a series of coordinated systems (e.g. cognitive, emotional, physiological) that are triggered by exposure to a sexual stimulus [1–4]. The cognitive system involves the integration of automatic and controlled attentional processing of sexual stimuli [2,3]. Specifically, attentional processing is thought to be a regulatory mechanism whereby dynamic changes in sexual arousal occur as a result of shifts in attention either towards or away from sexual cues [1–3]. Thus, sexual arousal is a product of attentional processing of sexual cues. Sexual arousal is thought to develop similarly in men and women; however, despite hypothesized similarities in the underlying processes, there are myriad gender differences in sexual response outcomes (e.g. sexual arousal, sexual desire, sexual concordance) [5–7]. Given that models of sexual arousal highlight the integral role of attention in the development of sexual outcomes, we examined gender differences in attentional processing of sexual cues as one potential mechanism underlying the observed gender effects in sexual responding in the literature.

One of the most notable gender differences in the sexual response patterns of men and women relates to the stimulus features (e.g. gender cues) that elicit a sexual response (for reviews see [8–10]). Gynephilic (i.e. sexual attraction to women) men's genital and self-reported sexual response patterns are gender-specific, such that they reliably exhibit a significantly stronger response to stimuli that correspond with their stated sexual preferences (i.e. preferred gender cues) compared with nonpreferred gender cues [11–19]. Androphilic (i.e. sexual attraction to men) women's genital response patterns are most often gender-nonspecific, such that there is no significant differentiation in sexual responses to preferred and nonpreferred gender cues [12,13,16,20–25].

Although the gender difference in specificity of sexual response is robust, it has largely been observed in studies using dynamic stimuli (e.g. audiovisual films or audio narratives), where the sexual activities and interactions change across the duration of the stimulus [12,13,16,20–25]. One exception to this body of research is a recent study that used a stimulus set that did not include the typical sexual context cues (i.e. no sexual activity, relationship cues or visible secondary sex characteristics). The researchers used static images of prepared sexual stimuli (erect penises and engorged vulvas) and observed for the first time gender-specific patterns of genital response in both gynephilic men and androphilic women [26]. Even though androphilic women's sexual response patterns tend not to be distinguished on the basis of gender cues, they have been shown to discriminate between stimuli describing conventional and masochistic sexual activities [27], and stimuli describing different relationship contexts (i.e. greater arousal to stories depicting interactions with strangers and long-term partners compared with friends) [21].

Researchers hypothesize that differences in attentional processing of sexual cues as a function of stimulus modality and content (e.g. sexual activity cues, relationship cues), might, in part, explain gender differences and similarities in specificity of sexual response [10,26]. Specifically, researchers have posited that the presence of sexual activity and relationship cues might activate greater attentional processing in women, including attention towards nonpreferred sexual targets, resulting in gender-nonspecific response patterns. By contrast, stimuli with fewer contextual features might result in weaker elaboration or greater attention towards preferred sexual targets contributing to gender-specific response patterns in androphilic women. Given the consistency with which men exhibit a gender-specific response pattern, attentional processing of preferred and nonpreferred targets may be less influenced by sexual contextual cues, such that men's attention may be biased towards preferred gender targets regardless of the presence of sexual activity cues or the stimulus modality used [10,26].

Data from the visual attention literature provides evidence to support the hypothesis that attentional processing of sexual cues is influenced by the features within a stimulus [28]; however, we are limited in the conclusions we can draw in terms of visual processing of preferred and nonpreferred gender cues due to differences in the study designs and stimuli used. Using a forced attention paradigm—where two single-target images are simultaneously presented and compete for attention—Dawson & Chivers [29] observed differences in attentional biases to preferred and nonpreferred gender cues in androphilic women and gynephilic men, and these differences were dependent on the stage of attentional processing (initial versus controlled) assessed. Specifically, they observed a gender difference in initial orienting towards preferred and nonpreferred targets, such that gynephilic men oriented significantly more quickly to preferred female targets (i.e. gender-specific), whereas androphilic women oriented similarly quickly to both preferred male and nonpreferred female targets (i.e. gender-nonspecific). By contrast, measures of controlled attentional engagement (total fixation duration (TFD) and total fixation count) were significantly greater for preferred targets compared with nonpreferred targets, providing evidence of gender-specificity of controlled visual attention in both genders. Dawson & Chivers [29] also observed significant positive relationships between measures of controlled attention and self-reported sexual attraction—a product of attentional processing—consistent with models of sexual response. Similar patterns of initial and controlled visual attention and self-reported attraction (SRA) have been observed in other studies that have used stimuli void of sexual activity, relationship and nonsexual contextual cues (i.e. background), in women with varied sexual attractions [30] and in men [31,32].

Other studies examining visual attention biases to preferred and nonpreferred gender cues have used static still images that include sexual activity cues (e.g. male–female dyads engaged in sexual activity) [33,34]. In addition to the sexual targets and sexual activity cues, the stimuli also included background features that could capture attention (i.e. nonsexual contextual cues). In contrast to the studies above that used single-target images with no sexual activity cues and no background/contextual cues [29–31], studies using images of couples engaging in sexual activity have consistently observed a gender difference in gender-specificity of controlled attention, similar to studies of genital response using stimuli depicting sexual activity. Men preferentially attend to the preferred female target (i.e. gender-specific pattern), whereas women distribute their attention more evenly across both the preferred male target and the nonpreferred female target (i.e. gender-nonspecific pattern) [33,34]. The findings from studies using decontextualized static images versus more contextualized static images provide initial support for the Spape *et al*. [26] assertion that attentional processing of sexual cues is influenced by the presence of sexual activity cues and that this may be one factor contributing to gender effects observed in studies of sexual arousal.

Gender differences in attentional processing of nonsexual contextual regions of stimuli (e.g. background) might also explain differences in the sexual response patterns of men and women. That is, greater attention towards nonsexual regions would likely result in weaker and perhaps less differentiated genital response patterns. Rupp & Wallen [34] observed a gender difference in the degree to which the background attracted visual attention, such that women using oral contraceptives spent significantly more time attending to the background of the static image of a couple engaged in sexual activity than did naturally cycling women and men. Using similar stimuli, however, Lykins *et al*. [33] observed that men and women spent similar amounts of time attending to nonsexual contextual cues depicted within the stimuli. Thus, it remains unclear the degree to which nonsexual contextual cues attract attention, if this is gendered, and if this relates to specificity of sexual response.

To our knowledge, only one study has examined attentional processing of sexual cues using dynamic video stimuli comparable to what is typically used in studies of sexual arousal. Consistent with studies using static images of couples engaged in sexual activity, Tsujimura *et al*. [35] observed a gender difference in specificity of visual attention. In line with all previous studies using stimuli depicting couples, men looked significantly longer at preferred targets. Interestingly, rather than a nonspecific pattern of visual attention observed for static images of coupled sex, women looked significantly longer at nonpreferred targets (i.e. gender-specific but in the direction incongruent with their self-stated sexual preferences). Women also spent more time viewing nonsexual cues (e.g. background) than did men. One limitation to the Tsujimura *et al*. [35] study is that the authors limited their analyses to a 40-s segment in the middle of the 240-s stimulus; thus, it remains unclear whether or not the pattern of results are specific to the 40-s segment chosen for analyses.

From the extant data we can conclude that the presence of sexual activity cues results in gender-nonspecific visual attention in androphilic women and gender-specific visual attention in men, and that the nature of these sexual activity cues (dynamic versus static) further influences attention, but only in women, such that nonpreferred sexual targets are preferentially attended to. Taken together, eye-tracking studies using stimuli depicting single targets versus couples and static still images versus dynamic videos, provide support for the hypothesis that preferred and nonpreferred gender cues are processed differently at the initial and controlled attention stages, and that these differences are influenced by the stimulus modality (static versus dynamic) and cues (gender, sexual activity, nonsexual contextual) depicted. The extant data also suggest that attentional processing of stimuli is gendered, and that the stimulus modality and cues within a stimulus may have a stronger influence on women's patterns of initial and controlled attention, than they do on men's. Given the importance of attention in the proliferation of sexual response, these differences in attentional processing may be one mechanism that explains the varied patterns of self-reported and genital sexual response observed in studies using different stimulus modalities to elicit sexual arousal [12,13,16,20–25].

### 1.1. The current study

The purpose of this study was to examine how stimulus modality (static versus dynamic) and cues (gender, sexual activity and nonsexual contextual) influence attentional (i.e. gaze) and elaborative (i.e. SRA, self-reported arousal) processing of sexual stimuli in women and men. Owing to differences in incentivization of sexual cues, we hypothesized that visual attention towards preferred and nonpreferred targets would be affected by stimulus modality and that this effect would differ for men and women. For men, we predicted greater attention towards preferred gender cues at both the initial and controlled stages of attentional processing, regardless of stimulus modality. For women, we predicted that initial attention would be similarly attracted by preferred and nonpreferred gender cues (i.e. gender-nonspecific), followed by greater controlled attention towards preferred gender cues (i.e. gender-specific pattern). For dynamic video stimuli, we predicted that the presence of sexual activity cues would result in greater attention towards nonpreferred sexual targets, due to increased opportunity for elaborative processing of the dynamic sexual cues. To further explore gendered attentional processing of sexual stimuli, we predicted a gender difference in gaze towards nonsexual/background regions of the dynamic film stimuli, such that women would attend more to nonsexual/background regions than would men. In addition to exploring the role of stimulus modality on visual attention, we also examined the effects on SRA to the male and female targets (outcomes of attentional processing). We predicted that both men and women would report gender-specific patterns of attraction but that these effects would be stronger in men, regardless of stimulus modality. Finally, to demonstrate the relationship between attention and sexual outcomes proposed in models of sexual response, we examined the relationships between initial and controlled attentional processing of sexual cues and SRA to the targets. We hypothesized that both attentional capture (i.e. initial attention) and attentional engagement (i.e. controlled attention) during static and dynamic stimuli would be positively related to SRA, and that this effect would be moderated by gender. We also hypothesized that degree of specificity of visual attention during dynamic stimuli would be positively related to self-reported sexual arousal in men, and negatively related to self-reported sexual arousal in women.

## 2. Methods

### 2.1. Participants

Participants were recruited through the Queen's University Undergraduate Psychology Subject Pool. To be eligible to participate, individuals were required to be over 18 years of age, able to read and write English fluently, have normal or corrected-to-normal vision, and have previously viewed sexually explicit media. Thirty men and 46 women were included in the study, all of whom reported exclusive or predominantly sexual attractions to the other gender based on the sexual attraction item from the Kinsey scale [36,37]. Replicating previous studies [29,30], no significant differences were detected in gaze times to male and female targets as a function of sexual attraction. As such the current results are collapsed across sexual attraction group. Demographic information for the sample can be found in table 1. Compensation for participation was 2% course credit. All the procedures were approved by the General Research Ethics Board at Queen's University.

**Table 1.** Demographic information for androphilic women and gynephilic men.

	gynephilic men *M* (s.d.) *n* (%)	androphilic women *M* (s.d.) *n* (%)
age	18.8 (1.3)	18.9 (1.2)
relationship status		
single	22 (73%)	25 (54%)
dating	8 (27%)	21 (46%)
sexual attraction		
exclusively other-gender attracted	24 (80%)	32 (70%)
predominantly other-gender attracted	6 (20%)	14 (30%)
ethnicity		
European	21 (70%)	29 (63%)
Asian	2 (7%)	6 (13%)
Hispanic	1 (3%)	1 (2%)
First Nations	0 (0%)	1 (2%)
other	6 (20%)	9 (20%)
highest education completed		
high school	4 (13%)	9 (20%)
university (attending/completed bachelor's degree)	26 (87%)	37 (80%)
employment		
full-time student	24 (80%)	31 (67%)
part-time	2 (7%)	11 (24%)
unemployed	3 (10%)	2 (4.5%)
other	1 (3%)	2 (4.5%)
normal vision		
yes	18 (60%)	33 (72%)
corrected-to-normal	12 (40%)	13 (28%)
hormonal contraceptive		
yes	—	30 (65%)
no	—	16 (35%)

## 3. Materials

### 3.1. Experimental stimuli

The stimuli used were images and videos taken from freely accessible Internet websites. The images had been used in previously published studies [29,30] and depicted nude male and female targets provocatively positioned with their aroused genitals clearly visible. All images were standardized to minimize the influence of low-level features on attention (for a full description, see [29]).

A total of 40 image trials were presented in a forced attention paradigm. Each trial involved two images presented simultaneously (one male target and one female target) in opposing corners of the screen (top left/bottom right or top right/bottom left) for a 10-s duration. Image location was balanced across trials, with the distance to each other and the centre fixation held constant. The picture pairings were matched with respect to their width and were equidistant from the centre fixation point.

The video stimuli included 28 different videos of 20-s duration each presented without sound depicting a variety of sexual activities (nude exercise, masturbation, coupled sex) and targets (male, female, male–male, female–female and male–female). To facilitate comparisons with the image stimuli, only the four films depicting male–female coupled intercourse are examined in this paper. The video presentations were randomized and divided into two blocks, such that each block contained two exemplars of male–female intercourse. To resemble the forced attention paradigm used for the static still images, the videos were intentionally selected to ensure that the spatial positioning of the male and female targets were distinct and equally represented for the duration of the 20-s video (i.e. the faces and bodies of the actors did not overlap and each target captured a similar proportion of the screen). The point of genital contact was clearly visible; however, it was not included in the analyses. There were two videos depicting rear-entry penile–vaginal intercourse, and two depicting penile–vaginal intercourse with the woman on top position. The camera angle was consistent across films (i.e. the targets were filmed from side on so that the features and responses of each target were clearly visible and each target was equally represented in the scene). This ensured that when participants were fixating on the female target they could not simultaneously fixate on the male target and that attention would not be biased by other confounding factors. Each video had a different background; however, the backgrounds across the videos depicted similar content (e.g. bedroom furnishings). The videos were matched for quality, size and were visually matched for luminance. Unfortunately, it was not possible to perfectly control for luminance in the videos in the same way that was done for the static still images.

### 3.2. Apparatus

All eye movements were measured using a Tobii T60 eye tracker and Tobii Studio^TM^ 2.2 software. The Tobii T60 is a contact-free, remote-sensor, eye-tracking system that uses an infrared camera to measure bright and dark pupil tracking. The system is compatible with both eyeglasses and contact lenses. All stimuli were presented on the 17-inch monitor at a resolution of 1280 × 1024 pixels with a temporal resolution of 60 Hz.

### 3.3. Post-stimulus self-reported attraction and arousal

Following the presentation of each image pair or video, participants were asked to rate how sexually attracted they were to the man and to the woman in the stimulus, separately, using a 7-point scale ranging from 0 (*not at all sexually attracted*) to 3 *(moderately sexually attracted)* to 6 (*very sexually attracted*). Following each video, participants were asked to rate how sexually aroused they felt using a 7-point scale ranging from 1 (*not at all sexually aroused*) to 4 (*moderately sexually aroused*) to 7 (*very sexually aroused*).

### 3.4. Questionnaires

Participants completed three questionnaires during the course of the study: a baseline questionnaire, an in-laboratory questionnaire and a post-laboratory questionnaire. Within the in-laboratory questionnaire participants provided demographic information and their sexual history (table 1). Sexual attractions were assessed using the Kinsey scale [36,37].

## 4. Procedure

Participants were welcomed to the laboratory and received a verbal explanation of the experimental procedures by a trained research assistant. Written informed consent was provided by each participant prior to participation in the study. Participants first completed a baseline questionnaire to assess their sexual interests and behaviours in the three days prior to their laboratory session using an iPad. Once completed, the research assistant rejoined the participant in the room to set up the eye-tracking component of the experiment. The research assistant positioned the participant at a distance of approximately 60 cm from the eye-tracking monitor. Prior to viewing the images and videos, participants were instructed that it was important that they look at each sexual target because they would be asked to rate their degree of sexual attraction towards the male and female target in each of the images and videos presented.

The stimuli were presented in three blocks. All participants viewed the stimuli in the same predetermined order. Previous research has demonstrated that attentional biases to preferred and nonpreferred cues do not change across block [29]. The first block included the 40 image trials, with an additional eight practice trials (depicting clothed male and female targets) to give participants the opportunity to familiarize themselves with the task. The remaining two blocks included the 28 video trials, 14 per block. Participants were given the opportunity to rest after each of the three blocks. The eye tracker was calibrated using a 9-point calibration procedure prior to each block (i.e. a minimum number of three times throughout the experiment).

Prior to each trial, a small fixation point appeared on the centre of the screen for 2 s to ensure that all participants were looking at the same point of the screen at the beginning of each trial. Following the fixation point, two images appeared and remained on the screen for 10 s, or a video was presented for 20 s. After the stimulus presentation, two questions appeared one at a time in the same order each time. The first asked ‘How sexually attracted were you to the man?' and the second asked ‘How sexually attracted were you to the woman?'. Participants responded on a scale from 0 to 6 using the computer mouse. After the video stimuli, participants answered an additional question: ‘How sexually aroused do you feel?'. Participants responded on a scale from 1 to 7 using the computer mouse.

Once the eye-tracking component of the study was finished, participants completed a second questionnaire assessing a variety of individual difference variables using an iPad. Once completed, participants were debriefed about the components of the study that they had completed up until that point and were told that they would receive an email in three days containing a link to the final component of the study—the post-laboratory questionnaire. Once all tasks had been completed participants received a second debriefing form containing details about the full study, were granted course credit, and were thanked again for their participation.

## 5. Data preparation and analysis

### 5.1. Eye movements

Eye movement data were recorded with Tobii Studio^TM^ 2.2 software. Fixation identification was filtered using an algorithm that identifies fixations and removes saccadic eye movements. The algorithm measures the distance between neighbouring gaze points and calculates the eye movement velocity for all the eye movements sampled for each individual. Raw data points are assigned to the same fixation if the velocity remains below a set threshold or are assigned to a new fixation when the velocity rises above this threshold (dispersal threshold of 30 pixels corresponding to 0.9° and a minimum temporal duration of 100 ms).

#### 5.1.1. Regions of interest

Two regions of interest (ROI) were designated for each image pair (male ROI and female ROI). Initial attentional biases were measured by the latency to first fixate (LTFF) on the male or female ROI (in seconds) for each trial. LTFF scores were then averaged across all valid trials (i.e. trials where gaze was fixated on the centre fixation point for the 1 s prior to the stimulus presentation) to create two variables of initial attention—one for the male ROI and the other for the female ROI. To capture controlled attentional biases, the TFD—the total amount of time spent (in seconds) looking—was calculated for each of the two ROIs for each image trial and averaged across all valid trials yielding two variables of controlled attention. Raw LTFF, TFD and SRA values as a function of stimulus modality and target type are presented in table 2.

**Table 2.** Raw gaze data for androphilic women and gynephilic men as a function of stimulus modality and sexual target gender.

	gynephilic men *M* (s.d.)					androphilic women *M* (s.d.)				
	image stimuli			video stimuli		image stimuli			video stimuli	
sexual target	LTFF	TFD	SRA	TFD	SRA	LTFF	TFD	SRA	TFD	SRA
preferred	0.91	5.54	3.04	12.16	3.73	1.28	4.98	2.23	6.79	1.89
	(0.39)	(1.24)	(1.02)	(2.38)	(0.99)	(0.51)	(1.08)	(1.02)	(1.89)	(1.04)
nonpreferred	1.85	2.36	0.27	4.57	0.28	1.24	3.48	0.84	9.67	1.07
	(0.66)	(1.16)	(0.52)	(1.69)	(0.55)	(0.46)	(1.00)	(0.91)	(2.18)	(1.05)

ROI designation for the videos needed to account for the dynamic nature of the stimuli and the inclusion of nonsexual contextual features. Each 20-s film was binned into 20 1-s epochs prior to designating the ROIs. Within each 1-s epoch, a unique ROI was created for the male and female targets, and the nonsexual contextual (i.e. background) region. Doing so ensured that the ROI corresponded with the target's movement throughout the 20-s film. LTFF was not computed for the video stimuli because both targets were positioned in the central region of the screen rendering initial orienting meaningless because the participant's gaze was already directed to the centre fixation. Instead, for each video, TFD towards the male target was summed across the 20 1-s bins, TFD towards the female target was summed across the 20 1-s bins and TFD towards the nonsexual region was summed across the 20 1-s bins. These summed scores were then averaged across the four video exemplars, yielding three TFD variables for male, female and nonsexual contextual ROIs, respectively.

#### 5.1.2. Latency to first fixate gender-specificity index

To assess the degree of specificity in initial orienting towards preferred and nonpreferred sexual targets for the static images, LTFF to the male and female ROIs were computed into an index score. To do so, we subtracted the LTFF towards the nonpreferred target from the LTFF towards the preferred target. Preferred target was determined by self-reported androphilia or gynephilia based on responses to the Kinsey scale [36,37]. For interpretability, we then multiplied the index score by −1 so that positive index scores would reflect faster orienting towards preferred targets (i.e. a gender-specific pattern congruent with SRA), whereas negative scores reflect slower orienting towards preferred targets (or faster orienting to nonpreferred targets). Index scores not significantly different from 0 reflect a gender-nonspecific pattern.

#### 5.1.3. Total fixation duration gender-specificity index

To assess the degree of specificity in controlled attention towards preferred and nonpreferred sexual targets for the static images and dynamic videos, TFD to the male and female ROIs were computed into an index score. To do so, we subtracted the TFD towards the nonpreferred target from the TFD towards the preferred target. To facilitate comparisons across stimulus modalities for the degree of gender-specificity of controlled attention, it was necessary to account for the different stimulus presentation lengths (10 s versus 20 s). To standardize across the static image and dynamic video stimuli, we divided the index score by the stimulus length (10 s and 20 s for images and films, respectively). Positive index scores reflect greater attentional engagement with preferred targets (i.e. a gender-specific pattern congruent with SRA), whereas negative scores reflect greater attentional engagement with nonpreferred targets. Index scores not significantly different from 0 reflect a gender-nonspecific pattern.

### 5.2. Self-reported attraction and arousal ratings

#### 5.2.1. Self-reported attraction gender-specificity index

The index score for the SRA variable was calculated by subtracting the attraction rating to the nonpreferred target from the attraction rating to the preferred target. Self-reported arousal for each of the four videos was averaged to get an average self-reported arousal score for each participant.

### 5.3. Data analysis

To be included in the analyses, participants needed to have at least 20 usable image trials (i.e. 50% usable data). Of note, participants included in the analyses mean number of usable trials was much greater than 50% (*M*_usabletrials_ = 33.8, s.d. = 5.1 out of a possible 40 trials). Degree of gender-specificity of initial attention was examined by subjecting the LTFF Gender-specificity Index Score to a one-way analysis of variance (ANOVA) with participant gender as the between-subjects factor.

To examine the effect of stimulus modality on controlled attention and SRA, each of the dependent variables (TFD gender-specificity Index; SRA gender-specificity Index) were subjected to a 2 (*Stimulus modality*: static, dynamic) × 2 (*Participant gender*: man, woman) mixed-model ANOVA. Significant interactions were further examined using Toothaker's mixed-model *t*-tests [38]. Toothaker's mixed-model *t*-tests maximize power by pooling the within- and between-subject error terms from the omnibus ANOVA.

For each of the moderated regressions models, one of the visual attention variables (either the LTFF or TFD Gender-specificity Index Score), Participant Gender and their interaction were entered into the model simultaneously to predict the SRA Gender-specificity Index Score or Mean Self-reported Sexual Arousal Score. Neither of the independent variables was centred because there were no issues with multicollinearity between the continuous independent variables and the dichotomous moderator. Significant interactions between the independent variable (LTFF or TFD) and the moderator (Participant Gender) were followed up using simple slopes analysis [39], with gender dummy-coded. This method enabled the strength of the relationship between the independent variable and dependent variable to be examined separately for androphilic women and gynephilic men.

## 6. Results

### 6.1. Gender-specificity of initial attention biases elicited by static stimuli

**Figure 1.**
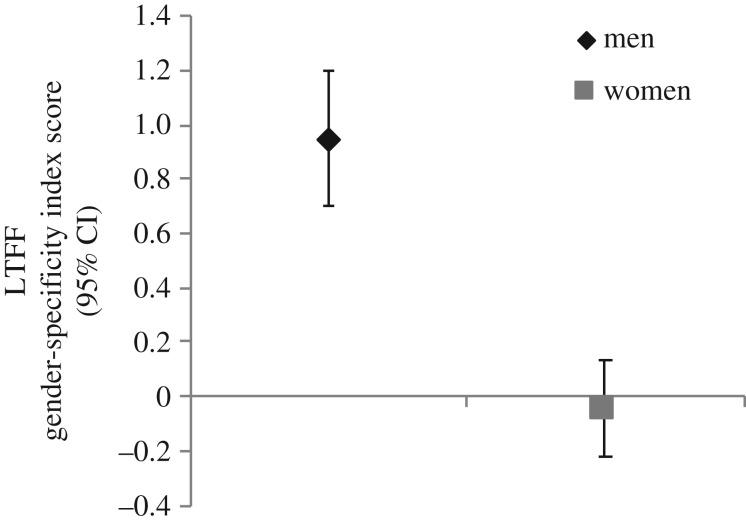
Androphilic women's and gynephilic men's latency to first fixation as an index score (preferred minus nonpreferred gender × −1) for static stimuli. Error bars represent 95% CI.

A one-way ANOVA using the LTFF Gender-specificity Index Score with Participant Gender as the between-subjects factor was used to examine gender differences and similarities in gender-specificity of initial attention. As seen in figure 1, there was a significant gender difference in initial attention, *F*(1,75) = 46.92, *p* < 0.001, *d* = 1.59, such that men exhibited a gender-specific pattern of initial attention towards preferred sexual targets (i.e. faster latency to first fixate; *M* = 0.95, s.d. = 0.66), whereas women did not differentiate between preferred and nonpreferred targets (i.e. similar latency to first fixate; *M* = −0.046, s.d. = 0.59).

### 6.2. Gender-specificity of controlled attention biases elicited by static and dynamic stimuli

**Figure 2.**
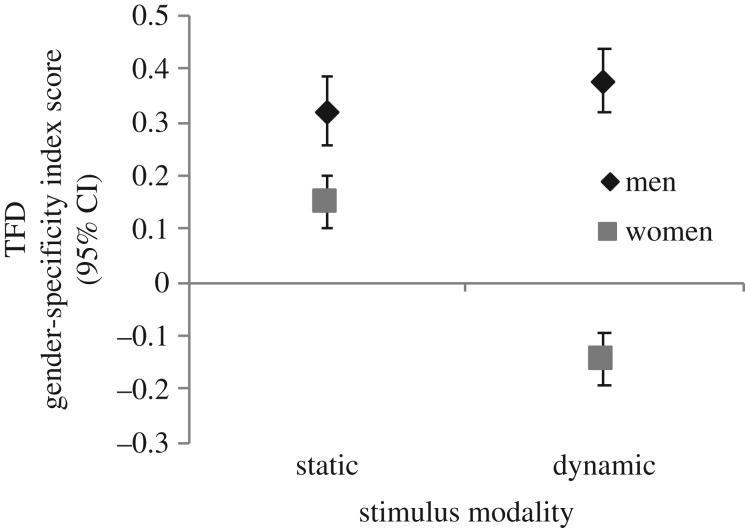
Androphilic women's and gynephilic men's TFD as an index score (preferred minus nonpreferred gender) for static and dynamic stimuli. Error bars represent 95% CI.

The 2 × 2 ANOVA using the TFD Gender-specificity Index Score results revealed a significant interaction between Stimulus Modality and Participant Gender, *F*(1, 74) = 79.52, *p* < 0.001, *η*_p_^2^ = 0.52 (figure 2). The degree of gender-specificity of men's visual attention was similar for static (*M* = 0.32, s.d. = 0.19) and dynamic (*M* = 0.37, s.d. = 0.18) stimuli, *t*(74) = −1.34, *p* = 0.18, *d* = 0.31. Stimulus modality did, however, significantly impact women's gender-specificity of visual attention, *t*(74) = 8.35, *p* < 0.001, *d* = 1.88. Women's controlled attention patterns were significantly gender-specific for static stimuli (*M* = 0.15, s.d. = 0.16), whereas they attended significantly longer towards nonpreferred sexual targets (*M* = −0.14, s.d. = 0.15) depicted in dynamic stimuli. In terms of gender effects, for both stimulus modalities, the degree to which men's visual attention differentiated between preferred and nonpreferred targets was greater than women's degree of differentiation, *t*(74) = 4.28, *p* < 0.001, *d* = 0.98, and *t*(74) = 13.17, *p* < 0.001, *d* = 3.19, for static and dynamic stimuli, respectively. The index scores on which these analyses are based control for stimulus length; however, examination of only the first 10 s of the dynamic stimuli produced a similar pattern of results.

### 6.3. Controlled attention towards nonsexual contextual cues during dynamic stimuli

To examine gender differences in the degree to which attention was attracted by nonsexual regions of the dynamic video stimuli, we used the TFD towards nonsexual contextual ROI score. An independent samples *t*-test revealed no significant difference between women (*M* = 3.44, s.d. = 2.58) and men (*M* = 3.27, s.d. = 2.10) in the time spent viewing nonsexual contextual regions of the video stimuli, *t*(74) = 0.29, *p* = 0.78, *d* = 0.07.

### 6.4. Gender-specificity of self-reported attraction elicited by static and dynamic stimuli

**Figure 3.**
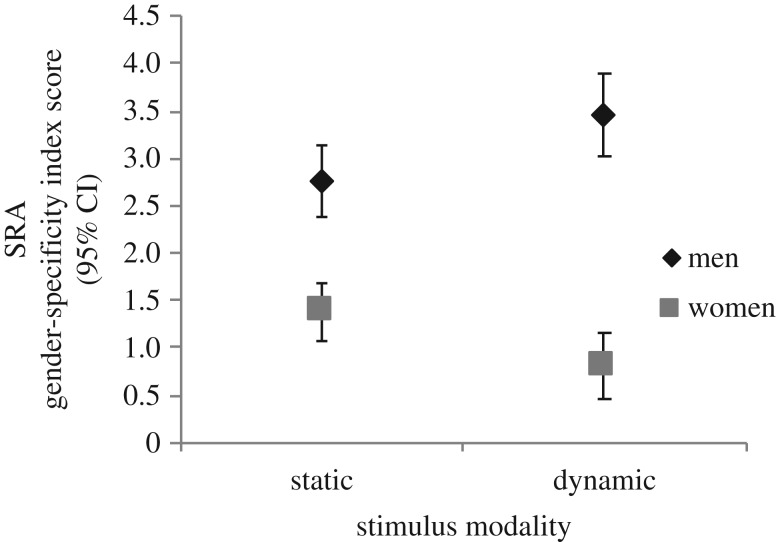
Androphilic women's and gynephilic men's SRA as an index score (preferred minus nonpreferred gender) for static and dynamic stimuli. Error bars represent 95% CI.

There was a significant interaction between Stimulus Modality and Participant Gender for the SRA Gender-specificity Index Score, *F*(1, 74) = 77.81, *p* < 0.001, *η*_p_^2^ = 0.51 (figure 3). Men's degree of gender-specificity of SRA was significantly impacted by stimulus modality, *t*(74) = −2.38, *p* = 0.02, *d* = 0.63, such that the degree of gender-specificity was weaker for static (*M* = 2.77, s.d. = 1.04) compared with dynamic (*M* = 3.45, s.d. = 1.12) stimuli. Stimulus modality significantly impacted women's degree of gender-specificity of SRA, *t*(74) = 2.45, *p* = 0.02, *d* = 0.50, such that women's degree of gender-specificity was stronger for static (*M* = 1.38, s.d. = 1.03) compared with dynamic (*M* = 0.81, s.d. = 1.23) stimuli. Additionally, men's degree of gender-specificity of SRA was stronger than women's for both static, *t*(74) = 5.31, *p* < 0.001, *d* = 1.34 and dynamic stimuli, *t*(74) = 10.11, *p* < 0.001, *d* = 2.22 (figure 3).

### 6.5. Relationships between initial and controlled visual attention biases and self-reported attraction during static and dynamic stimuli

**Figure 4.**
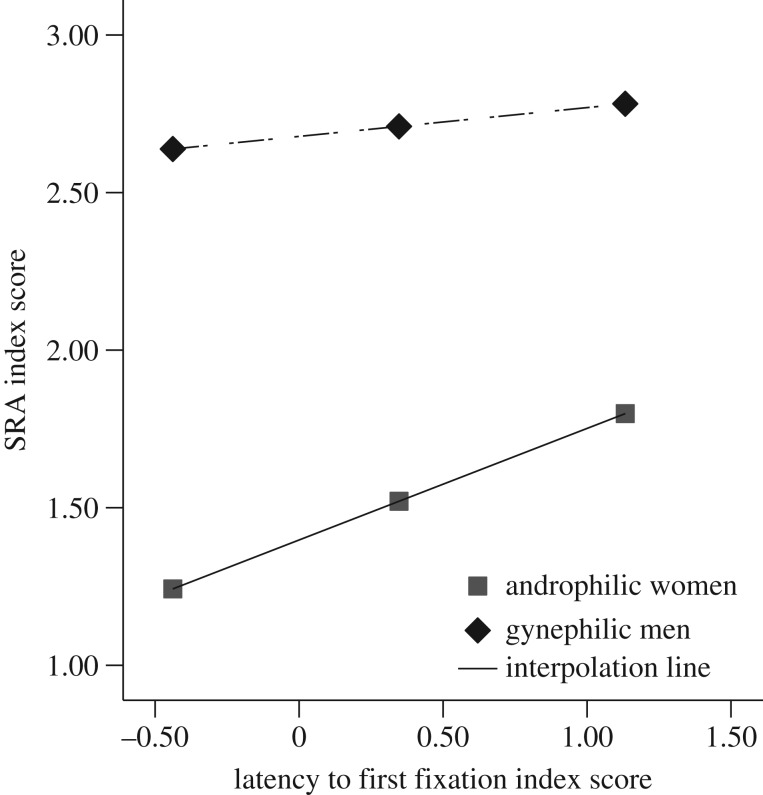
The relationship between initial attention and SRA (Gender-specificity Index Scores) during static still images. Gender did not moderate the relationship between initial attention and SRA; however, this figure depicts the positive relationship for both androphilic women and gynephilic men.

Moderated regression was used to investigate the strength of the relationship between initial orienting to preferred and nonpreferred targets (LTFF Gender-specificity Index Score) and SRA to preferred and nonpreferred targets (SRA Gender-specificity Index Score) elicited by static stimuli. As can be seen in figure 4, Participant Gender did not moderate the relationship between initial orienting and SRA, *B* = −0.26, *t*(72) = −0.67, *p* = 0.50. Because the moderation was not significant (i.e. the relationship between initial orienting and SRA was not significantly moderated by Participant Gender), simple linear regression was used to directly examine the relationship between initial visual attention and SRA, collapsed across Participant Gender. Initial orienting was a significant predictor of SRA, *B* = 0.69, *t*(74) = 4.18, *p* < 0.001, *R*^2^ = 0.19, such that greater degree of gender-specificity in LTFF was predictive of a stronger degree of differentiation in SRA.

**Figure 5.**
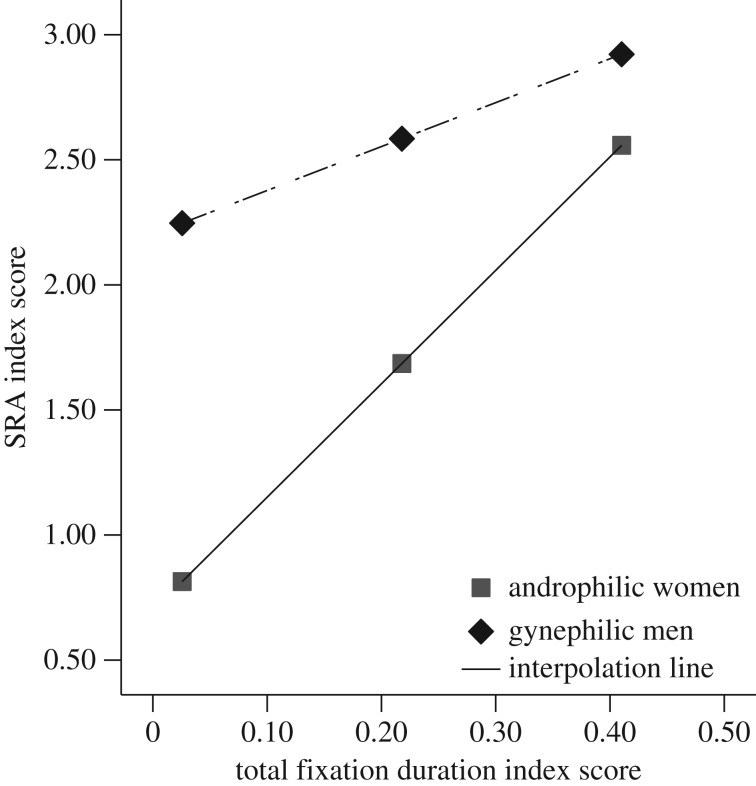
The relationship between controlled attention and SRA (Gender-specificity Index Scores) during static still images, moderated by gender.

The same analyses were used to examine the strength of the association between controlled attention (TFD) and SRA during static stimuli. Gender significantly moderated the effect of controlled attention on SRA for the static images, *B* = −2.78, *t*(72) = −2.46, *p* = 0.02. Simple slopes analysis revealed that, for androphilic women, the degree of gender-specificity of TFD significantly and positively predicted the degree of gender-specificity of SRA, *B* = 4.55, *t*(72) = 5.73, *p* < 0.001. For gynephilic men, the degree of gender-specificity of TFD significantly and positively predicted the degree of gender-specificity of SRA, *B* = 1.76, *t*(72) = 2.17, *p* = 0.03. Figure 5 depicts a stronger predictive relationship between controlled visual attention and SRA in women compared to men.

**Figure 6.**
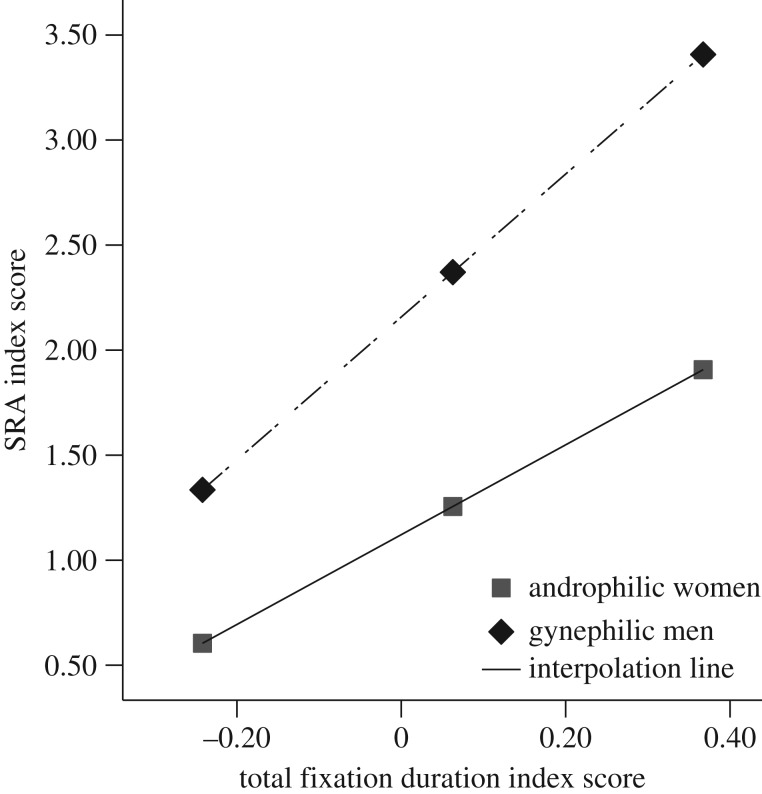
The relationship between controlled attention and SRA (Gender-specificity Index Scores) during dynamic stimuli. Gender did not moderate the relationship between controlled attention and SRA; however, this figure depicts the positive relationship for both androphilic women and gynephilic men.

A similar moderated regression model was used to investigate the relationship between controlled attention and SRA during dynamic stimuli. Participant Gender did not moderate this relationship, *B* = 1.26, *t*(72) = 0.80, *p* = 0.43, as can be seen in figure 6. Simple linear regression revealed that controlled attention was a significant predictor of SRA, *B* = 4.37, *t*(74) = 10.09, *p* < 0.001, *R*^2^ = 0.58, such that greater degree of gender-specificity in TFD was predictive of a stronger degree of differentiation in SRA.

### 6.6. Relationship between controlled visual attention biases and self-reported arousal during dynamic stimuli

An independent samples *t*-test revealed that men (*M* = 3.83, s.d. = 1.61) and women (*M* = 3.30, s.d. = 1.01) reported similar levels of arousal in response to the videos, *t*(44.03) = −1.58, *p* = 0.12, *d* = 0.40 (Levene's test for equality of variances was significant, so adjusted degrees of freedom are reported). Moderated regression was then used to investigate the relationship between controlled attention and self-reported arousal during dynamic stimuli. Participant Gender did not moderate this relationship, *B* = 3.33, *t*(72) = 1.85, *p* = 0.07. Simple linear regression collapsed across gender revealed that controlled attention was not a significant predictor of self-reported arousal, *B* = 0.56, *t*(74) = 1.14, *p* = 0.26, *R*^2^ = 0.13.

## 7. Discussion

The goal of this study was to examine how stimulus modality (static versus dynamic) and cues (gender, sexual activity and nonsexual contextual) influence attentional (i.e. gaze) and elaborative (i.e. SRA, self-reported arousal) processing of sexual stimuli in women and men. Replicating previous research using static images [29], we observed significant gender differences in initial and controlled attention towards preferred and nonpreferred gender cues. Attentional capture, as assessed by the latency to first fixate on either target, revealed that, for men, preferred sexual targets preferentially attract visual attention (i.e. gender-specific initial attention), whereas for women, preferred and nonpreferred targets attract attention similarly quickly (i.e. gender-nonspecific initial attention). By contrast, men and women each exhibited an attentional engagement bias (i.e. they fixated longer) towards their preferred sexual target. Taken together, these findings add to the growing body of the literature that attentional processing of preferred and nonpreferred gender targets differs depending on whether attentional capture versus attentional engagement is assessed, and that the effect of attentional stage is especially pronounced in women compared to men [29–31].

The use of dynamic stimuli enabled a preliminary investigation into how sexual activity and nonsexual contextual cues impact attentional engagement with sexual stimuli, and if these effects are also gendered. Consistent with previous research using images and videos of couples engaged in sexual activity, men's controlled visual attention was significantly greater to their preferred sexual target, whereas women attended significantly more to the nonpreferred female target [33–35]. Similar to Lykins *et al*. [33], we did not detect a gender difference in attention towards nonsexual contextual cues (cf. [34]). Rupp & Wallen's [34] sample of women were distinguished based on their hormonal contraceptive status, with women using oral contraceptives showing a bias towards attending to the background and contextual regions of the stimuli. It is possible that a visual attention bias towards nonsexual background cues is influenced by hormonal status as well as gender. Given the consistency with which images and videos depicting sexual activity between a man and a woman produce gender differences in specificity of controlled visual attention, it seems plausible to conclude that men and women differ in the ways in which they attend to complex sexual stimuli.

Models of sexual arousal posit that attentional processing of sexual cues contributes to sexual outcomes [1–4]. For both men and women, attentional processing of sexual cues was positively related to self-reported sexual attraction for static and dynamic stimuli, and these effects were stronger for controlled attention compared to initial attention. This effect is consistent with models of arousal that hypothesize that controlled attentional processes are stronger predictors of self-report outcomes because controlled attention is a better proxy for elaborative processing, whereas initial attention is more reflexive and predictive of physiological arousal [3]. Surprisingly, controlled visual attention was not predictive of self-reported arousal.

### 7.1. Gendered attentional processing of sexual stimuli

Gender differences in attentional processing of sexual stimuli are well replicated [29,32–35,40], yet explanations for these differences remain largely hypothetical. Below we discuss three potential sources of the gender difference in visual attention. First, gender effects in visual attention may arise because different features of the same sexual stimulus attract men's and women's visual attention (i.e. bottom-up processing) [28,41,42]. Second, men and women may adopt fundamentally different task-dependent attentional strategies when viewing sexual stimuli and this influences which features of the stimulus are attended to (i.e. top-down processing) [42–44]. And third, individual difference variables (e.g. hormonal status, sex drive, attitudes, sexual experience) may influence the salience of sexual stimuli and the attentional strategies employed [42,44,45]. We believe that all three contribute concomitantly to produce gender effects in attentional processing of sexual cues.

Within the emotional literature, visual attention has been shown to be captured by various types of stimuli, including specific regions of the stimuli that contain information, whether it be emotional, appetitive or aversive [e.g. 46–48]. Lykins *et al*. [40] established that sexual information is processed by the visual system differently than nonsexual information, such that attention is drawn to specific regions of sexual stimuli depending on the degree of sexual explicitness [33,34,40]. There is also a large body of evidence to support that regions of the body that signal mate quality and attractiveness (e.g. waist-to-hip ratio) also preferentially attract and sustain visual attention (reviewed in [49]). The current results extend these findings, such that the presence of dynamic sexual activity cues also influences bottom-up processing of sexual information. Sexual activity cues within more complex stimuli bias women's, but not men's attention towards their nonpreferred sexual target, as evidenced by the significant and large shift in degree of specificity for women (*d* = 1.88), compared with a nonsignificant and small shift in degree of specificity for men (*d* = 0.31). Contextual information relevant to the sexual salience of the stimulus (i.e. sexual activity cues) results in different patterns of attentional engagement with preferred and nonpreferred sexual targets compared to when these targets are presented individually. Thus, it seems reasonable to hypothesize that the gender difference typically observed for arousal patterns (reviewed in [10]) is more likely the result of gender differences in attentional processing of sexual contextual information rather than nonsexual contextual information (i.e. the background). The observation that men and women attended similarly to nonsexual contextual cues suggests that differences in specificity of arousal are not the result of men attending more to sexual cues eliciting a strong sexual response and women attending more to nonsexual contextual cues eliciting weaker and nonspecific responses across a range of sexual stimuli. Further, the degree to which specific stimulus features capture attention depends on the salience of these cues for the observer [43,44].

Top-down influences on attention include prioritizing information or cues based on their relevance/salience. Although we did not assess relevance or salience in this study directly, it could be argued that for our sample of androphilic women and gynephilic men, preferred sexual targets are more relevant and salient from an evolutionary standpoint than are nonpreferred sexual targets (for discussion, see [29,49]). There is growing evidence to suggest that task demands impact eye movement behaviour (reviewed in [44,50,51]). We used a motivated rather than free viewing task, where participants were instructed to look at both targets to facilitate ratings of sexual attraction and to report overall feelings of sexual arousal during the dynamic stimuli. This task demand would impact top-down processing of the sexual stimuli by influencing both the stimulus features that attract attention (preferred and nonpreferred targets), as well as the strategies men and women employ when viewing sexual stimuli based on the relevance/salience of the targets for the observer and to the task itself [44,50,51].

Women and men may adopt different attentional strategies when viewing sexual stimuli in order to augment their arousal. There is some evidence to suggest that men adopt more of an objectification or observer stance when viewing sexual stimuli by focusing their attention towards their preferred sexual target [52–55]. By contrast, women may be more likely to project themselves into a stimulus, identifying with the nonpreferred female target [52–55]. Men's gender-specific pattern of visual attention was congruent with their SRA, regardless of the stimulus modality used. This may reflect an objectification viewing strategy that relies more heavily on focusing on preferred sexual targets rather than projecting themselves into the stimulus; however, support for this hypothesis has not been found when examining patterns of arousal [52]. The observation that women's controlled attention was gender-specific during dynamic stimuli (but in the direction not congruent with SRA), may support the hypothesis that women's attention to nonpreferred sexual targets reflects a viewing strategy that augments arousal through identification with the target of the same gender. Specifically, attending to the female target may help women project themselves into the stimulus, which would lead to greater elaborative processing and increased arousal [52,54]. The static image task may not have elicited this same viewing strategy because the less intense stimuli may limit opportunities for elaborative processing. Although we cannot directly infer the viewing strategy men and women used, these findings provide indirect support for the hypothesis that men and women may adopt different viewing strategies in order to modulate their own sexual interest and arousal, and this effect might be more pronounced when task demands require participants to report on their arousal or degree of sexual attraction.

It is important to note, however, that there was variability in the degree of specificity for our dependent variables for both genders, thus, other factors not examined in the study (e.g. hormonal status, relationship status, sex drive, sexual sensation-seeking) may further influence visual processing of preferred and nonpreferred cues. Using cognitive reaction-time based tasks, Dewitte [2] observed that compared to women, men's attention was more easily drawn to sexual cues, especially when given opportunities for elaboration through longer stimulus presentation times. Men were also more strongly motivated to approach sexual stimuli and were better able to inhibit their attention towards sexual cues. We observed similar results using eye tracking. Men were faster to detect sexual cues than were women, attended longer to sexual cues, and reported greater sexual attraction, but not arousal. It is possible that the combination of high appetitive drive towards detecting and elaborating on preferred cues coupled with the strong ability to inhibit attention to nonpreferred cues may have also contributed to the gender-specific patterns observed in men. By contrast, nonspecificity in women's initial attention to static images, and gender-specificity in their controlled attention to dynamic stimuli that are incongruent with their sexual attraction, may be the result of less appetitive drive to preferred cues and/or less inhibition to nonpreferred cues.

Other trait-level individual difference variables appear to further influence visual attention towards sexual cues. For example, attitudinal factors, such as homonegative attitudes result in general avoidance of nonpreferred sexual targets, especially in men [56]. More general dispositional factors also influence gaze. Bradley *et al.* [57] observed a significant negative relationship between disgust and gaze time towards sexual cues in women. Further, Tsujimura *et al*. [45] observed a significant negative relationship between social introversion and time spent viewing the sexual region of a stimulus in men. State-level factors, such as degree of sexual arousal may also reinforce attention strategies [58]. For example, when in a state of high arousal, the motivational salience of stimuli may shift with arousal in a positive feedback loop, resulting in certain stimulus features attracting attention preferentially. Of note, Jones [58] compared specificity of gaze in an arousal versus neutral induction condition and did not find an effect of arousal on the stimulus features (i.e. gender cues) that attracted attention in women; however, this does not mean that arousal state would not impact the viewing strategies employed by men and women.

An important consideration that we have not discussed is the possibility that the gender effects observed are not specific to sexual stimuli, but rather reflect broad gender differences in visual processing of information. Support for this hypothesis, as well as the three factors described above, comes from Moss *et al*. [44]. They demonstrated that bottom-up, top-down and individual difference factors work together to produce gender effects in visual attention to a variety of stimuli. Using stimuli of various categories (images from action films, romance films, wildlife documentaries, surrealist and nonsurrealist art), they observed significant gender differences in the features that attract attention (i.e. bottom-up influences). Irrespective of stimulus category, the authors observed a gender difference in specificity of visual attention for all stimuli depicting humans, such that all participants preferentially fixated on female targets, and this effect was more pronounced in women than men. To examine gender effects in top-down influences on visual attention, the authors instructed participants to complete three rating tasks (valence, potency and activity level) while viewing the images. All three of these rating tasks resulted in women adopting a fundamentally different visual search strategy compared to men, such that there was a reliable gender difference in eye movement behaviour. Women, on average, tended to be more exploratory in their gaze patterns, dispersing their gaze across the various stimulus regions by making more fixations to nonface locations, whereas men made less-frequent fixations and fixations were more concentrated to specific regions. Finally, the authors observed significant relationships between individual difference factors and gaze patterns. Men's and women's eye movement patterns were positively predicted by extraversion, perseverance and conscientiousness, whereas other personality factors uniquely predicted women's gaze (e.g. openness to experience and premeditation) and men's gaze (e.g. urgency).

Gaze patterns may then be gendered for all sorts of stimuli and that perhaps the effects observed for sexual stimuli reflect this broader gender difference. If women's attention is preferentially attracted to female targets, in general (see [10] for a discussion of nonsexual motivations behind women attending to female targets), and that regardless of instructional condition they adopt a visual search strategy that is more exploratory than men's, and if factors such as openness to experience are predictive of gaze patterns, then we might expect that women would produce a gender-nonspecific visual attention pattern. Further, if attention is requisite for sexual arousal, we would also predict that sexual arousal patterns would be similarly nonspecific or incongruent with sexual attraction. Future studies are needed to examine these interesting findings further and to test this hypothesis that gendered visual processing relates to the observed gender effects in sexual arousal.

### 7.2. Limitations and future directions

While the purpose of this study was to examine the effect of different sexual cues and stimulus modality on gendered visual attention, we would be remiss if we did not acknowledge that sexual activity cues and stimulus modality were conflated in our study. As such, the inclusion of an additional stimulus type (i.e. static images depicting couples engaging in sexual activity) would have provided a stronger test of our predictions and would have enabled us to disentangle whether it is the mere presence of sexual activity cues (that are not necessarily dynamic) or the dynamic nature of the stimulus, that influences visual attention. Although we did not include this stimulus category, the vast majority of previous research has used static images of couples engaged in sexual activity [33,34]. Thus, although a within-subjects comparison would provide the strongest test, we are confident in our conclusion that specificity of visual attention in women is influenced by the presence of sexual activity cues (in static and dynamic stimuli) and that this effect becomes more pronounced when dynamic stimuli are used (i.e. becomes gender-specific in the direction incongruent to SRA). Future studies should assess visual attention and physiological arousal concurrently using static and dynamic stimuli in order to further test models of sexual response and understand the potential attentional mechanisms contributing to patterns of gender-specificity and nonspecificity that have been observed in the literature.

If we accept that men's and women's attention is differentially influenced by stimulus features and that they adopt different attentional strategies when viewing the same sexual stimuli, then these gender differences have methodological implications for future research on patterns of sexual arousal. Researchers should be cognizant that the stimulus modality, sexual activity cues and overall complexity of the stimulus appears to impact women's attention to a greater degree than men's. When planning sexual psychophysiological studies, careful consideration should be given to the types of stimuli selected. For example, using different stimuli for men and women limits gender comparisons because the response patterns observed might be stimulus-specific, influencing bottom-up processing of sexual features. By contrast, using the same stimulus for men and women does not necessarily ensure that men and women will see the same features or that they will adopt the same viewing strategy, which will also influence response outcomes and gender comparisons. Further, if the same stimulus is used for both men and women, researchers should be mindful that this stimulus selected does not stack the deck, so to speak, in favour of eliciting a certain response pattern in either gender. Finally, the instructions that commonly accompany psychophysiological studies (e.g. ratings of various physiological and emotional states) likely influence the viewing strategies and stimulus features attended to. While these instructions are consistent for men and women, researchers should consider how these demand characteristics might influence attention patterns and subsequent response patterns.

## 8. Conclusion

This study adds to the growing body of literature that suggests that many aspects of visual attention are gendered. To this end, we observed significant gender effects on attentional processing of sexual stimuli and these effects were dependent on the stage of information processing (i.e. initial versus controlled), the stimulus modality used to elicit attention (i.e. static versus dynamic) and the specific cues within the stimuli (i.e. preferred and nonpreferred sexual targets, sexual activity cues, nonsexual contextual cues). In support of models of sexual response, attention and sexual outcomes (i.e. sexual attractiveness ratings) were significantly related; however, contrary to these models, the relationship between attention and self-reported arousal was not significant. Based on these findings, we propose that these gender effects result from differences in the stimulus features that attract attention, task-relevant demands and motivational state of the observer, as well as individual differences that influence gaze.

## Supplementary Material

Visual Attention and Self-report Data

## Data accessibility

Data are currently available as the electronic supplementary material.
